# A scoping review of burden of disease studies estimating disability-adjusted life years due to *Taenia solium*

**DOI:** 10.1371/journal.pntd.0010567

**Published:** 2022-07-06

**Authors:** Andrew Larkins, Mieghan Bruce, Carlotta Di Bari, Brecht Devleesschauwer, David M. Pigott, Amanda Ash

**Affiliations:** 1 Global Burden of Animal Diseases Programme https://animalhealthmetrics.org; 2 Centre for Biosecurity and One Health, Harry Butler Institute, Murdoch University, Perth, Australia; 3 School of Veterinary Medicine, Murdoch University, Perth, Australia; 4 Department of Epidemiology and Public Health, Sciensano, Brussels, Belgium; 5 Department of Translational Physiology, Infectiology and Public Health, Ghent University, Merelbeke, Belgium; 6 Institute for Health Metrics and Evaluation, Department of Health Metrics Sciences, School of Medicine, University of Washington, Seattle, Washington, United States of America; Seoul National University College of Medicine, REPUBLIC OF KOREA

## Abstract

**Background:**

*Taenia solium* is the most significant global foodborne parasite and the leading cause of preventable human epilepsy in low and middle-income countries in the form of neurocysticercosis.

**Objectives:**

This scoping review aimed to examine the methodology of peer-reviewed studies that estimate the burden of *T*. *solium* using disability-adjusted life years.

**Eligibility criteria:**

Studies must have calculated disability-adjusted life years relating to *T*. *solium*.

**Charting methods:**

The review process was managed by a single reviewer using Rayyan. Published data relating to disease models, data sources, disability-adjusted life years, sensitivity, uncertainty, missing data, and key limitations were collected.

**Results:**

15 studies were included for review, with seven global and eight national or sub-national estimates. Studies primarily employed attributional disease models that relied on measuring the occurrence of epilepsy before applying an attributable fraction to estimate the occurrence of neurocysticercosis-associated epilepsy. This method relies heavily on the extrapolation of observational studies across populations and time periods; however, it is currently required due to the difficulties in diagnosing neurocysticercosis. Studies discussed that a lack of data was a key limitation and their results likely underestimate the true burden of *T*. *solium*. Methods to calculate disability-adjusted life years varied across studies with differences in approaches to time discounting, age weighting, years of life lost, and years of life lived with disability. Such differences limit the ability to compare estimates between studies.

**Conclusions:**

This review illustrates the complexities associated with *T*. *solium* burden of disease studies and highlights the potential need for a burden of disease reporting framework. The burden of *T*. *solium* is likely underestimated due to the challenges in diagnosing neurocysticercosis and a lack of available data. Advancement in diagnostics, further observational studies, and new approaches to parameterising disease models are required if estimates are to improve.

## Author summary

*Taenia solium* is a parasitic worm of people and pigs. It is the most significant global foodborne parasite and causes neurocysticercosis, the leading cause of preventable human epilepsy in low and middle-income countries. Due to this significance, many people have attempted to estimate the burden of disease of *T*. *solium*. The disability-adjusted life year is the most common method for measuring burden of disease and combines the number of years of life lost with the number of years of life lived with disability due to an illness. This review found that many published studies probably underestimate the true burden of disease due to the challenges in diagnosing neurocysticercosis. If estimates are to become more accurate there must be further research into the epidemiology of *T*. *solium* and new approaches to disease modelling. Current estimates can be difficult to compare and a reporting framework for burden of disease studies may increase their utility by facilitating subsequent use and interpretation of different approaches.

## Introduction

*Taenia solium* is a zoonotic parasite commonly known as the pork tapeworm. It has been recognised as the most significant foodborne parasite in the world and is the leading cause of preventable epilepsy in low and middle-income countries (LMICs) [1–3]. Its complex lifecycle predominantly involves humans and pigs, with humans capable of acting as both definitive and intermediate hosts. As the definitive host, humans develop taeniosis after consuming raw or undercooked pork containing viable cysticerci. Humans with taeniosis host adult worms in their gastrointestinal tract and shed eggs or proglottids into the environment via their faeces, acting as a source of infection for others and themselves. Both pigs and humans may become intermediate hosts and develop cysticercosis after ingesting eggs or proglottids from human tapeworm carriers. Although taeniosis itself does not appear to result in significant morbidity, cysticercosis in humans may have major consequences [4]. The most significant of these is neurocysticercosis (NCC), which develops when cysts occur in a person’s central nervous system [5]. The most common sequelae due to NCC are epilepsy and seizures, which occur in approximately 80% of cases [6]. Definitively diagnosing a person with NCC can be a difficult task and requires advanced neuroimaging, brain or spinal cord biopsy, or in rare cases visualization of subretinal cysticerci [7]. These diagnostic methods are rarely available in the LMICs where NCC is endemic.

Neurocysticercosis patients incur morbidity and mortality due to the disease, whilst potentially losing income and becoming ostracised by their local communities [8,9]. Some pigs with porcine cysticercosis may present with seizures however, how often this happens is unknown. Even without clinical signs, porcine cysticercosis can lead to pig values being markedly reduced or carcasses being condemned at slaughter, significant issues for smallholder farmers [10,11]. Given the visible impact of NCC on people’s lives and the high prevalence of *T*. *solium* in some communities [12], multiple analysts have looked to estimate the burden of disease (BoD) as a means of summarising the impact of *T*. *solium* and evaluating health interventions. Disability-adjusted life years (DALYs) have rapidly been adopted as the predominant human BoD measure by considering both morbidity and mortality in a single metric. The process of completing BoD studies that apply DALYs generally involves at least three fundamental steps: the creation of a disease model that describes the course of illness and related health states, collection and adjustment of data, and the DALY calculation itself.

### A brief background to disability-adjusted life years

Discussion surrounding the principles of DALYs have been ongoing since they were first published in 1994 [13–15]. Whilst appearing conceptually simple, as the sum of the years of life lost (YLL) and years of life lived with disability (YLD) due to a given cause [16,17], DALYs in reality require analysts to consider multiple underlying parameters that may significantly impact their result and its application (Table 1).

**Table 1:** Basic DALY considerations.

DALY parameter		Description	Impact and relevance
YLL	Life table	Life tables provide the life expectancy of a person at different ages and, possibly, for different sexes.	Aspirational life tables allow for comparison between countries, time periods, and population subgroups; however, they do not reflect the observed life expectancy. National life tables reflect current (period) life expectancies; however, they introduce a negative correlation between mortality rates and YLL weights, and limit comparisons.
YLD	Perspective	An incidence perspective considers all future health states that contribute to disability, whereas the prevalence perspective only considers health states that are experienced in a given time period.	Whilst the two approaches are both valid, an incidence perspective will usually provide a higher estimate of YLD by including future duration of disability beyond the current period.
	Duration	The future length of time that a disability is experienced.	Only required when taking a prevalence perspective to YLD. Representative data may be difficult to source.
	Disability weights	Different health states inflict different levels of disability or morbidity on a person. A DW of zero implies no disability, whilst a DW of one is equivalent to death.	The choice of disability weights will change YLD results. Different disability weights are not incorrect and may reflect differences in societal health values or methods.
General	Time discounting	DALYs incurred in future time periods have their original values discounted.	The inclusion of time discounting reflects a preference that will differ between scenarios.
	Age weighting	Certain ages are weighted higher than other ages.	Weighting the value of one person’s life over another due to their age presents an ethical quandary that is not easily answered and represents a social preference.

Years of life lost are those years that a person does not experience due to their premature death. They are calculated by examining the expected age limit of a person and subtracting their age of death. The age limit of a person is usually taken from a model life table that is based on demographic modelling. The choice of life table holds practical implications for YLL results and presents an ethical quandary relating to how the unexperienced age limit should be set and valued [14,18,19]. The application of an aspirational life table representing an ideal scenario, or a national life table based on observed data, is a frequent decision that must be made in BoD studies. Whilst a national life table may provide BoD estimates that reflect the current situation and reality in a country, their use means that the DALY estimate cannot be compared with other countries, sub-populations, or time periods [19]. If they were to be compared, countries with low life expectancies may appear to have fewer YLL per death than countries with higher life expectancies. Diseases occurring in low life expectancy countries may therefore appear less significant than diseases in high income countries. These are unethical and irrational results on a global scale, and present limitations in comparing results over time or across population sub-groups.

Years of life lived with disability are the DALY’s measure of morbidity and reflect the number of healthy years that a person loses due to illness. Disability weights (DWs) are the DALYs primary method for relating morbidity and mortality. Each health state related to an illness is assigned a DW ranging from zero (perfect health) to one (equivalent to death). This DW, combined with the number of cases, provides the basis for YLD and allows for the DALY’s combination of morbidity and mortality. The choice of DWs to be used will clearly have an impact on resulting YLD values and DWs have been developed using a variety of different methods in different populations [20,21]. Before any YLD calculation can be completed, it must be decided if cases and their respective morbidity will be measured with an incidence or prevalence perspective. An incidence perspective to YLD counts incident cases and includes health losses that may occur in future years. Incident YLD are calculated as a product of the incident cases, their disability weight (DW), and the future duration of disability. Prevalent YLD consider prevalent cases and their current disability. They are simply the product of prevalent cases and their DW. The symptomatic period of a health state may be considered for acute or intermittent causes; however, it is capped at a single year. This incidence versus prevalence consideration is not required for YLL as all deaths are viewed as incident [16,22].

Age weighting and time discounting are two similar tools that are applied to the DALY to reflect societal or organisational preferences. When applied, age weighting reflects the preference that a year of life lived at one age is worth more than another. This preference is not necessarily rational but may be based on the potential of life, the economic productivity of different age groups, or other societal values [13]. Similarly, time discounting reflects the preference that the benefits and costs of today are valued more than those in the future. When applied to DALYs, this means that DALYs experienced in future years represent a lower burden than those experienced today. Given that age weighting and time discounting reflect social preferences with no single correct or easily resolvable answer, original GBD work provided estimates with and without age weighting and time discounting [13]. Since 2010, IHME have attempted to simplify their GBD results by choosing to remove both age weighting and time discounting from their methods [17].

These fundamental methodological choices relating to DALYs are the foundation of any BoD study. They are required even without considering additional complexities presented in disease models, sensitivity analysis, uncertainty, and missing data. If the basic process of the DALY calculation is not adequately described or differs between studies, the comparison of different estimates becomes limited. The comparison of a combined morbidity and mortality metric is arguably the DALY’s most powerful application, and its removal may eliminate some of its appeal over other BoD metrics.

### Rationale and objectives

*Taenia solium*, particularly in the form of NCC, can be difficult to diagnose and as a neglected tropical disease its burden is largely felt in LMICs. The few national DALY studies for NCC provide estimates that range from 0.25–9.00 DALYs per 1,000 people in different locations, without considering the uncertainty intervals of the estimates [23]. Given the nature of *T*. *solium*, the relatively recent development of DALYs, and the variety of methods applied to BoD studies in other areas [24–27], there is a need to identify and summarise the first cohort of studies that have estimated the BoD due to *T*. *solium* using DALYs. The objective of this review is to investigate the methodologies that have been applied, with the goal of answering the following research questions:

- Which disease models have been applied?
- Where have data been sourced?
- How have DALYs been calculated?
- How have sensitivity analysis, uncertainty, and missing data been handled?
- What are the key limitations that have been presented?

## Method and materials

The general approach of this review follows the preferred reporting items for systematic reviews and meta-analyses extension for scoping reviews [28] (S1 Checklist). The review protocol was not registered prior to completion.

### Search method

Peer-reviewed studies of any language, published between 01 January 1991 and 14 August 2021, were considered eligible for review. As the DALY metric was introduced in 1994 by Murray [16], this date range was expected to capture all relevant publications. PubMed, Web of Science, and SCOPUS online databases were queried using English terms (S1 Protocol). Grey literature and pre-print publications were not considered. Additional publications from the WHO FERG and IHME GBD groups were included by searching their websites and examining the references from included studies. These two groups are well known in the burden of disease field and they have produced comprehensive burden estimates for a vast number of diseases. Given the breadth of their work, relevant publications from these organisations may not list all the diseases for which they have produced estimates. Consequently, such publications may not have been captured in the search terms and warrant their addition through other means. Attempting to capture these publications in the main search would have increased the number of studies that required screening to an unreasonable level. The most recent search was completed on 18 August 2021.

### Study selection, handling, and synthesis

Search results were downloaded from each source and uploaded to Rayyan for assessment [29]. Duplicate studies were removed before the remaining studies were screened by title and abstract then full text by a single reviewer (AL). Studies were excluded if they did not estimate the BoD due to *T*. *solium* in humans using DALYs, did not describe their methods for calculating the BoD, or repeated the results of previous studies. This final criterion ensured that large studies with multiple publications were only represented once in the review. Data from included studies were entered into a standardised workbook as they appeared in-print. Data captured included details relating to publication, disease models, DALY methods and results, uncertainty, sensitivity and missing data, and general narrative (S1 Protocol).

Disease models of *T*. *solium* were categorised as either direct, attributional, or transitional. Direct disease models obtain disease occurrence data that directly reflects the health state of interest, for example obtaining the number of deaths recorded as NCC from a government death register. Attributional models utilise data on a broader health state, such as epilepsy, and apply an attributable fraction to estimate the specific health state of interest, in this case NCC-associated epilepsy. Transitional models use the overall incidence of an agent or health state, such as *T*. *solium* or NCC, and apply a probability of progressing to different health states, such as epilepsy or death [30].

Data sources for key variables were recorded and had their reference and study populations categorised by location to assess if extrapolation was required. For DALY calculations, the application of discount rates and age weighting was noted. The life table used for YLL calculation was extracted. For YLD, the general perspective was classified as incident or prevalent [22,31]. In some cases, the perspective was not explicitly stated and had to be inferred from the relevant methods. Also recorded were the sequelae considered, source of DWs, and if severity levels and co-morbidity adjustments were applied. Sensitivity analysis, uncertainty, and missing data were assessed by comparing the methods used in each study. In this review, we consider sensitivity analysis to be focussed on variable importance analysis, whilst scenario analysis is considered as a method of capturing uncertainty. The key limitations stated in each publication were considered, with a particular focus on noting themes relating to the difficulty in diagnosing NCC, poor data availability, and underestimation.

## Results

**Fig 1:**
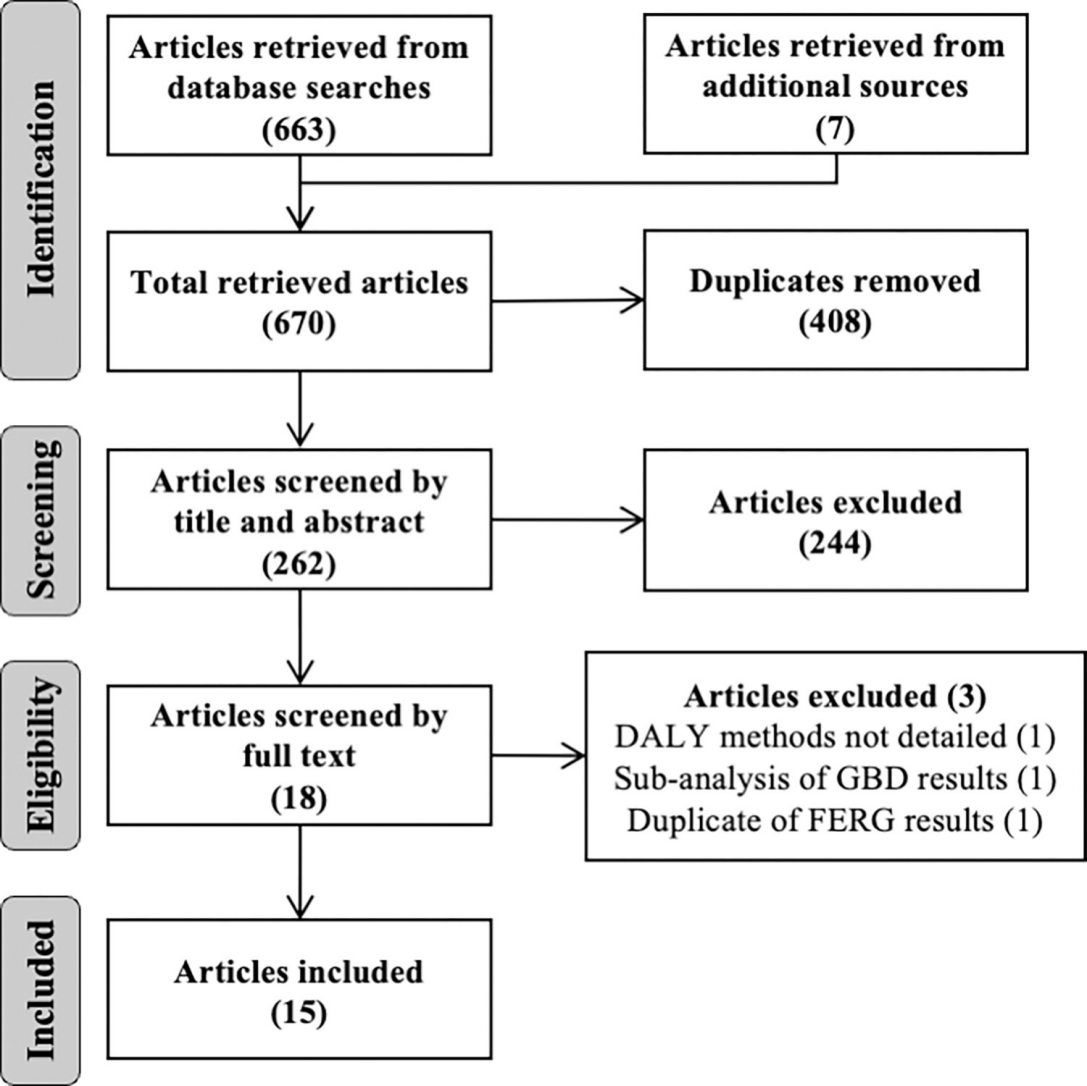
Flow diagram of study selection.

A total of 670 entries were retrieved from online databases. In addition, seven studies from FERG and GBD were identified and included in the review process. After removal of duplicates, screening, and eligibility assessment, 15 studies were included for review. Of the three studies excluded during the eligibility phase of the review, one did not detail the methods for calculating the DALY estimate presented [32], one analysed previously published GBD results [33], and one analysed previously published FERG results [34] (Fig 1). Of the 15 studies included, eight focussed on national or sub-national burdens in different countries with three studies from Africa [35–37], three from Asia [11,38,39], and two from South America [40,41]. The remaining seven studies were global estimates produced by FERG or GBD [42–48]. The purpose of almost all studies was to estimate the BoD of *T*. *solium* to inform public health planning, whilst one study measured the BoD to evaluate a specific One Health intervention in Laos [11].

### Disease models

All studies included in the review considered only the burden of NCC and did not include other *T*. solium-related illnesses. Six out of eight national or sub-national studies applied an attributional approach to the disease modelling for both sequelae and deaths due to NCC. These studies estimate the occurrence of sequelae before attributing a fraction of this to NCC and transitioning these cases to death or additional sequelae [11,35–38,40]. Coral-Almeida and collaborators [41] also estimated cases using an attributional model for sequelae; however, it was the only national or sub-national study to directly estimate the number of deaths. Singh et al. [39] on the other hand applied a direct approach to sequelae, utilising two door-to-door neuroimaging studies to estimate cases of NCC in people with epilepsy, before transitioning a proportion of these cases to death. The six GBD studies consider the population-at-risk of NCC and take an attributional approach to NCC cases, with the proportion of epilepsy attributable to NCC based on meta-analysis. Deaths due to NCC however, are directly modelled on vital registration and surveillance data from endemic countries [46]. The FERG applied a purely attributional approach by utilising the epilepsy envelope from GBD 2010 [2,47]. A fraction of the envelope was attributed to NCC based on meta-analysis and corrected for each country’s population-at-risk [49].

### Data sources

Data for the disease models were frequently sourced from observational studies. In six of eight national and sub-national studies, the occurrence of epilepsy was sourced from singular observational studies [11,36,37,39–41]. The geographic source of these parameters was often extrapolated from another location or smaller population [11,35,37–40]. Devleesschauwer et al. [38] applied meta-analysis to combine estimates from multiple studies, whilst Trevisan et al. [35] created a uniform distribution bound by the lower and upper limits of studies in an attempt to account for uncertainty and extrapolation. Similarly for the proportion of epilepsy due to NCC, only one national or sub-national study was able to apply the results from the same study population [36]. Again Devleesschauwer et al. [38] combined multiple estimates from within Nepal using meta-analysis, whilst Trevisan et al. [35], Coral-Almeida et al. [41] and Okello et al. [11] created uniform distributions bound by the lower and upper limits of Tanzanian, Ecuadorian, and nearby Asian studies, respectively. Bhattarai et al. [40] was the only national study to use a global meta-analysis to parameterise the proportion of epilepsy due to NCC [50]. Case fatality rates for national estimates were sourced from sub-national studies [35,39], government data [38], and WHO country-level data [40]. Where direct methods were applied, Coral-Almeida and colleagues [41] sourced the number of deaths from the national death registry which were attributed to NCC using the tenth revision of the international classification of diseases (ICD-10) code for cysticercosis of the central nervous system (B69.0). The GBD studies rely on vital registration and surveillance data from endemic countries to inform their cause of death models and include deaths reported as other forms of cysticercosis due to *T*. *solium* using ICD-10 codes B69.0–B69.9. For their non-fatal estimates, GBD attribute the occurrence of epilepsy to NCC based on a meta-analysis of available literature [46]. The primary parameter of the FERG model beyond the GBD epilepsy envelope is the proportion of epilepsy attributable to NCC, which was estimated using a separate meta-analysis to GBD [2,50].

### DALY calculations

All sub-national or national studies, with the exception of Trevisan et al. [36], calculated DALYs using an incidence perspective for YLD (Table 2). In each of these studies, the prevalence of epilepsy was converted to incidence [51]. This contrasts with the GBD estimates which have applied a prevalence perspective to DALYs since cysticercosis was first included in GBD 2010 [17]. The application of discount rates and age weighting is less consistent with different combinations applied (Table 3). Both FERG and GBD estimates utilise neither technique; and since 2014, five of six national or sub-national studies have followed suit [11,36] or presented estimates using multiple scenarios for discounting and age weighting [35,38,39]. The GBD studies have proven to be the source of most lifetables for YLL calculations, and all DWs and durations for YLD relating to epilepsy (Table 2). The GBD programme continues to update their lifetables and since being led by IHME, have produced two versions of DWs [52,53]. In terms of duration, the GBD estimates now take a prevalence perspective to YLD and do not require a future duration parameter. All incident-based studies relied on the future duration of epilepsy from the 1990 GBD study [54], albeit often cited as sourced from Praet et al [37]. With respect to comorbidity, only the GBD series, and FERG estimate by proxy of the GBD epilepsy envelope, consider comorbidity when calculating YLD.

**Table 2:** A summary of YLL and YLD parameters.

Region	Study	YLL	YLD				
		Life table	Perspective	Sequelae	Severity	Duration	Disability weights
Global	GBD series^a^ [42–48]	GBD	Prevalence	Epilepsy	Yes	–	GBD
National	Coral-Almeida et al., 2020 [41]	CDW	Incidence	Epilepsy; migraine	Yes; No	[37]^b^	[45]
	Singh et al., 2017 [39]	GBD	Incidence	Epilepsy	Yes	[37]^b^	[37]^b^
	Trevisan et al., 2017 [35]	Not stated	Incidence	Epilepsy	Yes	[54]	[53]
	Devleesschauwer et al., 2014 [38]	GBD; CDW	Incidence	Epilepsy	Yes	[37]^b^	[37]^b^
	Bhattarai et al., 2012 [40]	CDW	Incidence	Epilepsy; severe chronic headache	Yes; Yes	[54,62]	[37,54,82]^b^
Sub-national	Okello et al., 2018 [11]	Not stated	Incidence	Epilepsy	Yes	[54]	[52]
	Trevisan et al., 2018 [36]	GBD	Prevalence	Epilepsy; migraine; tension type headache	Yes; No; No	–	[52, 53]
	Praet et al., 2009 [37]	CDW	Incidence	Epilepsy	Yes	[54]	[54]

CDW: Coale-Demeny West model life tables, GBD: Global Burden of Disease series,–: not applicable

^a^This includes GBD studies from GBD 2010 [48] onwards and the FERG 2015 study [47]

^b^Praet et al, 2009 [37] is frequently cited as the source, though itself cites that the parameters used are taken from GBD 1990 [54].

**Table 3:** A summary of time discounting and age weighting decisions.

Region	Study	Time discounting	Age weighting
Global	GBD series^a^ [42–48]	No	No
National	Coral-Almeida et al., 2020 [41]	Yes	No
	Singh et al., 2017 [39]	Yes & no^b^	Yes & no^b^
	Trevisan et al., 2017 [35]	Yes & no^b^	Yes & no^b^
	Devleesschauwer et al., 2014 [38]	Yes & no^b^	Yes & no^b^
	Bhattarai et al., 2012 [40]	Yes	Yes
Sub-national	Okello et al., 2018 [11]	No	No
	Trevisan et al., 2018 [36]	No	No
	Praet et al., 2009 [37]	Yes	Yes

^a^This includes GBD studies from GBD 2010 [48] onwards and the FERG 2015 study [47].

^b^These studies provided estimates with and without time discounting and age weighting.

When considering the sequelae that contribute to YLD, twelve out of fifteen studies consider only epilepsy and stratify by severity and/or treatment using GBD strata. The remaining three studies included migraine, migraine and tension type headaches, or severe chronic headaches (Table 2). The method for calculating sequelae other than epilepsy is different in each of these studies. In Ecuador, an attributional approach was taken to the population prevalence of epilepsy and migraine, respectively, using data from case-control studies [55–57]. In Tanzania, Trevisan and collaborators [36] provided a fraction of NCC-associated epilepsy cases with addition of burden due to tension-type headaches or migraine based on local data [58]. Bhattarai et al. [40] was the first study to include additional sequelae in the form of severe chronic headaches. A decision-tree method was used to combine their estimate of the number of NCC-associated cases attending neurology clinics with the proportion of cases with severe chronic headaches, correcting for the proportion seeking treatment. This was also the only study that was able to stratify additional sequelae by severity.

### Sensitivity, uncertainty, and missing data

All models applied stochastic techniques to account for uncertainty in parameter precision and presented their results as 95% intervals. Four of the eight national or sub-national studies included scenario analysis as a method to reflect uncertainty in epidemiological parameters [37,39] or different preferences for time discounting and age weighting [35,38,39]. Four of the eight studies completed sensitivity analysis using regression techniques for variable importance [11,35,36,40]. The occurrence of epilepsy, proportion of epilepsy attributable to NCC, and the case fatality rate of NCC were parameters that consistently affected model output [11,35,36,39,40]. Neither FERG nor GBD estimates present sensitivity analysis for their NCC models. The primary method for handling missing data in national or sub-national studies was to apply data from another population. More sophisticated methods for handling missing data were present in FERG and GBD studies where missing data may be present in many countries across the globe. The FERG approach to missing data for most diseases was to apply a Bayesian random effects model; however, in the case of NCC, epilepsy data were sourced from GBD 2010 estimates and imputation had already occurred [49]. The GBD studies also impute missing data using Bayesian methods with the major difference in approach being the inclusion of covariates and fixed effects based on model performance [17,59–61].

### Key limitations

Apart from the GBD studies, all studies provide some commentary on the key limitations of modelling efforts. Given that, in general, GBD studies provide a less detailed discussion on their disease-specific models due to the scale of their modelling efforts, this possibly reflects no published discussion rather than no limitations. A lack of available data was an issue raised in all studies with discussion and seven of these nine studies commented they have likely underestimated the BoD due to this limitation; however, none were able to correct their estimates for underestimation [2,35–37,39–41].

## Discussion

### Disease models and data sources

The results from this review reveal a relatively consistent approach to disease modelling for the burden of *T*. *solium* where NCC is the only outcome considered. The attributional method that has been frequently applied to the disease models is a currently a necessity due to the diagnostic challenges of NCC. However, many of the studies that BoD estimates might rely on are unable to conclusively address the question of causality in their study populations due to this same challenge. Consequently, most BoD estimates are required to assume that the population attributable fraction of epilepsy in their population is the same as what has been found in the few locations where epilepsy and NCC have been studied together. In studies where both deaths and cases of NCC are based on the attribution of epilepsy, this assumption affects both YLL and YLD estimates. If direct cause of death data is applied, only YLD results will be impacted by the assumed attributable fraction. In these studies, YLL is instead impacted by the quality of cause of death data and these data also reflect the difficulty in measuring the occurrence of NCC. Coral-Almeida and colleagues [41] were the only national or sub-national study to use direct cause of death data and acknowledge this limitation. They further discuss that poorer areas without access to advanced diagnostics may not have their burden realised using this method. Given that *T*. *solium* is primarily a neglected tropical disease occurring in under resourced communities, the diagnostic challenges of NCC present a significant constraint on all current BoD estimates.

These key observational studies that BoD estimates rely on are even further limited when considering sequelae beyond epilepsy. There are only a small number, and they are primarily case-control studies of varying quality based in health facilities or small populations [55,56,58,62]. As a result, BoD studies attempting to include additional sequelae in their estimates have been inconsistent in their approach. This difficulty in quantifying the occurrence and association of sequelae due to NCC has led to many studies likely underestimating the true BoD, a common discussion theme identified in this review. Additional sequelae are an important consideration and will increase current BoD estimates. Whilst commonly reported symptoms are an essential starting point in understanding NCC sequelae [6], it is important to consider the relative occurrence of these symptoms in the wider population. Although a diagnostic and logistical challenge, additional case-control or cohort studies comparing sequelae in both NCC patients and non-NCC patients could provide further insight into the relative burden of NCC. Whilst likely to be minor, the inclusion of other *T*. *solium* outcomes beyond NCC, such as taeniosis and muscular cysticercosis, may also increase current BoD estimates.

Headaches are one sequelae that has the potential to increase the burden of NCC, given that they affect approximately one-third of patient and headache disorders were the 15^th^ leading cause of DALYs in 2019 [6,46]. Unfortunately, a comprehensive systematic review of the clinical manifestations of NCC was unable to describe the types of headaches due to NCC [6]. This is an important consideration given that the DWs for different types of headaches varies significantly [63]. If headaches are to be included, how they are modelled and the choice of DWs must be carefully examined and reflect the nature of NCC headaches and their treatment patterns. Given the intermittent nature of headaches, correction for the symptomatic period is required, even under a prevalence perspective to YLD, as noted in GBD methodology [46]. Further research into the association and occurrence of headaches and NCC, along with other sequelae is an important gap to be addressed.

A recent study in Uganda was published after this review had completed its data collection and analysis appears to be the first national estimate that includes headaches and considers their symptomatic duration [23]. This study was also the first to provide local NCC data to GBD’s DisMod II and “generate an internally consistent, complete and age-stratified set of epidemiological parameters for NCC epilepsy” [23]. The application of DisMod II to generate local parameters, particularly the duration of epilepsy, is promising and potentially removes one of the limitations identified in this review where studies have relied on an outdated duration parameter. Whilst promising, DisMod II or any other model are still only as reliable as the data that are provided to them, and do not alleviate the fundamental difficulty in measuring the occurrence of NCC.

### DALY calculations

The division between incident and prevalence perspectives identified in this review should not be seen as a detriment of the DALY metric, rather it reflects the context and purpose of each study. Just as incidence and prevalence may both be valid choices when measuring disease frequency. Both DALYs perspectives are equally valid and provide two different perspectives on disease burden [17,31]. The purpose of a BoD study and the application of its results must be considered when deciding on the perspective and the other DALY parameters discussed above. These are important methodological choices, and one must be particularly careful before directly comparing different DALY estimates as the results may be fundamentally different. It is for this reason that this review focussed on methodological choices rather than the study results themselves. It hoped that this review may enable subsequent informed comparisons of the results from different estimation processes. An example of one such pitfall that decision-makers could easily make, would be comparing the cost per DALY of multiple interventions without understanding their basic perspective on YLD or other DALY parameters. Those interventions that took an incidence approach would most likely, by their definition, have their cost-effectiveness inflated compared to those that took a prevalence approach [64]. Whilst this pitfall has not been identified in this review, with only the one study evaluating cost-effectiveness as cost per DALY [11], it should be considered when planning and analysing future *T*. *solium* studies and is relevant to any disease.

By taking an incidence perspective many of the national and sub-national studies in this review have been required to include two additional steps in their DALY calculations: converting prevalence to incidence and including future duration in the final YLD equation. Given current NCC diagnostics, the occurrence of NCC obtained from observational studies has been, and will be for the foreseeable future, based on the prevalence of epilepsy. The studies in this review commonly divided prevalence by the duration of illness to calculate incidence. It should be remembered that this conversion assumes that the population is stationary and additional modelling is often required to fulfil this assumption if the conversion is to be accurate [65]. Secondly, the future duration of illness must also be included in incident YLD calculations. Estimating the duration of any disease or health state is difficult at the best of times, even in well-planned observational studies. As a result, they are often limited to hospital-based populations in high-income countries and NCC BoD studies have been required to rely on the duration of epilepsy from the 1990 edition of GBD [54]. These two additional steps in calculating incident YLD have the potential to increase error and uncertainty in estimates. As many BoD studies are focussed a single point in time, it is understandable that an incident approach is the common choice and it should not be forgotten that all BoD estimates are driven by epidemiological data with analysts pragmatically using what data is available [19]. Nevertheless, studies should consider if the requirements of an incidence perspective will adversely affect their results and investigate methods that may account for this, such as the use of DisMod II or other statistical models [23,59].

### Sensitivity, uncertainty, and missing data

Bayesian methods, sensitivity analysis and scenario analysis are all commonly n applied to account for uncertainty and variability when estimating for the burden of *T*. *solium*. The variables with the greatest impact on results were those related to the basic epidemiology of NCC [11,35,36,39,40]. This highlights the need for improved diagnostics, further observational studies, and new approaches to parameterising disease models if BoD estimates are to become more certain. Missing data in BoD studies can be considered missing with respect to time or space for three main reasons: it has not been found by the analyst; it simply does not exist; or the quality of data make it unusable. This review found that in most cases, the perfect dataset for a *T*. *solium* estimate does not exist and all studies have been required to combine data sources of differing quality and quantity relating to disease occurrence and association.

Meta-analysis was another analytic tool that was applied to generate summaries from multiple observational studies and fill data that were missing. Meta-analysis has been particularly useful in providing a summary of the proportion of epilepsy attributable to NCC in endemic countries [50]. The application of such summaries beyond their original study populations is currently required; however, it may not be entirely appropriate given their geographic coverage. When considering the proportion of epilepsy attributable to NCC, the FERG meta-analysis was able to include only 12 studies from ten countries [50]. More recently, Gripper and Welburn included 25 studies from 11 countries [66]. When flexible diagnostic criteria were applied, Debacq and collaborators identified 14 studies from 10 countries [67]. This lack of fundamental epidemiological studies is particularly prominent in Asia, where only India is represented in any of the meta-analyses. The most recent GBD meta-analysis was completed for their 2015 edition and included 30 data sources from 16 countries, although the details of these sources were not stated [46]. *Taenia solium* is considered endemic in over 75 countries [68] and the current necessity to extrapolate data from a relatively small selection of these is potentially a significant limitation of current BoD estimates, particularly given that specific cultural and behavioural risk factors may play a significant role in NCC epidemiology [69,70].

### Limitations of this review

The limitations of this review lie primarily within its search strategy, review process, and choice of data to be collected. The search strategy and review process were based on database searching and published methods. Any studies that have not been published and the single study without sufficient methodological detail have been missed [32]. The search was conducted using English search terms and studies published in other languages will have also been missed. The use of only a single reviewer has the potential to introduce bias into the review process. The use of a standardised data charting workbook, the mostly quantitative nature of variables targeted, small number of studies expected to be included, and experience of co-authors hopes to have limited the impact of any reviewer bias. Given that there is no standard for reporting burden of disease studies, the selection of data to be collected in this review reflect the authors experience and previous work on the topic [19,23,24,27,71]. Additional methodological details relevant to BoD studies have not been addressed and include methods relating to age-standardisation, adjustments for comorbidity, and handling of missing data. These discussions are beyond the scope of this disease-specific review and are addressed by others in a wider context applicable to all BoD studies [15,17,59–61,72–75].

### Future estimates

This review illustrates that there is consistency in the overarching approach taken to estimating the BoD due to *T*. *solium*. This is not necessarily a positive attribute, with many of the studies in this review relying on the same parameters, extrapolating similar studies, and calling for improved *T*. *solium* data if more accurate and precise estimates are to be provided. Further observational studies, novel diagnostic tools, and increased access to neuroimaging are required, if parameters such as the occurrence, duration, and case-fatality of NCC and its related sequelae are to be better understood. These developments will take time, resources, and may never be achieved in some locations. As a result, we encourage BoD analysts to focus on: 1) How they may be able to apply additional modelling techniques to utilise the data they have available [23,59,76]; 2) How they can present their BoD methods in a transparent and complete manner so that comparisons between studies can be made more readily and their limitations highlighted and addressed.

The application of the Guidelines for Accurate and Transparent Health Estimates Reporting and studies that examine methodological choices are the beginning of a discussion that may help to improve BoD reporting [71,77]. The ideal outcome of such discussion would be the development of a readily accessible BoD reporting standard. Such a standard may allow studies and their estimates to be compared more easily and increase their utility. Consistency in reporting of BoD studies is not limited to *T*. *solium* and applies across all causes of disease. It is important that comparisons between diseases are made using relatively level principles and potential discrepancies due to methodology are identified. Studies focussed on *T*. *solium* are unlikely to provide any global direction on how BoD studies should age weight or time discount; however, they are the only arena in which the specifics of *T*. *solium* will be discussed. With the interim lack of standards, those conducting BoD studies should consider how their methods can be presented in not only a reproducible manner, but also a flexible manner, so that parameters may be altered to allow for further comparisons between studies. This issue is pertinent to this review, as one of the few relevant studies with access to neuroimaging had to be excluded because there was no description of DALY methods [32]. Limited detail may be appropriate for descriptive measures of disease frequency and association, as these are essentially unchanged since their inception. The DALY is a more recent measure that has the potential to take multiple forms and is not purely descriptive, through its inclusion of parameters that relate to social values and preferences [14,16,78].

This review has focussed solely on the methods for estimating DALYs due to *T*. *solium*. As DALYs do not account for the monetary cost of disease in humans or animal health impact they represent only one component of the complete burden of disease. Many of the national and sub-national studies included in this review also include the monetary burden on NCC patients and pig producers to provide a more complete assessment of the burden of disease [11,35–37,39]. Another promising metric that has had limited application to date is the zoonotic DALY (zDALY), which looks to relate the DALY and a new non-monetary measure of animal losses into a single value that may more completely capture the societal burden of zoonotic diseases [11,79]. With the development of the Global Burden of Animal Diseases programme [80,81] it is hoped that the BoD in humans and animals can be linked on a larger scale to further describe the complete burden.

## Conclusion

Estimating the BoD is an essential activity for public health actors across the globe and is a field that can help prioritise, monitor, and evaluate health systems. This review has illustrated the complexities associated with calculating DALYs and the need for standardised reporting. The current burden of *T*. *solium* is likely underestimated due to the difficult nature of diagnosing NCC and a lack of observational studies. Future BoD studies must look for new ways to make the most of their available data and continue to highlight their needs and methodological choices. Burden of disease studies are essential if progress is to be made towards understanding the true impact of *T*. *solium* and solidifying the rationale for investment in *T*. *solium* control. The authors of this review are aware of multiple *T*. *solium* studies that will provide new BoD estimates and it is hoped that this review can encourage those studies to produce consistent reporting of BoD estimates.

## Supporting information

S1 Checklist Prisma-ScR checklist. (PDF)

S1 Protocol Data collection workbook. (XLSX)
